# A Review of Biomechanical Gait Classification with Reference to Collected Trot, Passage and Piaffe in Dressage Horses

**DOI:** 10.3390/ani9100763

**Published:** 2019-10-03

**Authors:** Hilary M. Clayton, Sarah Jane Hobbs

**Affiliations:** 1 Sport Horse Science, 3145 Sandhill Road, Mason, MI 48854, USA; 2 College of Veterinary Medicine, Michigan State University, East Lansing, MI 48824, USA; 3 Centre for Applied Sport and Exercise Sciences, University of Central Lancashire, Preston PR1 2HE, UK; SJHobbs1@uclan.ac.uk

**Keywords:** horse, kinematics, duty factor, aerial phase, ground reaction force, inverted pendulum mechanics, spring-mass mechanics

## Abstract

**Simple Summary:**

This paper reviews the biomechanical classification of diagonally coordinated gaits of dressage horses, specifically, collected trot, passage and piaffe. Each gait was classified as a walking gait or a running gait based on three criteria: limb kinematics, ground reaction forces and center of mass mechanics. The data for trot and passage were quite similar and both were classified as running gaits according to all three criteria. In piaffe, the limbs have relatively long stance durations and there are no aerial phases, so kinematically it was classified as a walking gait. However, the shape of the vertical ground reaction force curve and the strategies used to control movements of the center of mass were more similar to those of a running gait. The hind limbs act as springs with limb compression increasing progressively from collected trot to passage to piaffe, whereas the forelimbs show less compression in passage and piaffe and behave more like struts.

**Abstract:**

Gaits are typically classified as walking or running based on kinematics, the shape of the vertical ground reaction force (GRF) curve, and the use of inverted pendulum or spring-mass mechanics during the stance phase. The objectives of this review were to describe the biomechanical characteristics that differentiate walking and running gaits, then apply these criteria to classify and compare the enhanced natural gait of collected trot with the artificial gaits of passage and piaffe as performed by highly trained dressage horses. Limb contact and lift off times were used to determine contact sequence, limb phase, duty factor, and aerial phase duration. Ground reaction force data were plotted to assess fore and hind limb loading patterns. The center of mass (COM) trajectory was evaluated in relation to changes in potential and kinetic energy to assess the use of inverted pendulum and spring-mass mechanics. Collected trot and passage were classified as running gaits according to all three criteria whereas piaffe appears to be a hybrid gait combining walking kinematics with running GRFs and COM mechanics. The hind limbs act as springs and show greater limb compression in passage and piaffe compared with trot, whereas the forelimbs behave more like struts showing less compression in passage and piaffe than in trot.

## 1. Introduction

Horses move naturally through a range of speeds by transitioning between different gaits when it is energetically beneficial [1], as a means of reducing limb forces [2] or to preserve gait stability [3]. Based on spatiotemporal gait descriptors, horses typically walk at slow speeds, trot at intermediate speeds, and canter/gallop at fast speeds. In ponies, the transition speeds are around 2 m/s from walk to trot [1] and 4–5 m/s from trot to gallop [1,2]. However, the *DMRT3* nonsense mutation allows the use of ambling gaits at intermediate speeds, and has a favorable effect on speed capacity in trot or pace allowing these gaits to be retained at high speeds rather than transitioning to a gallop [4]. The *DMRT3* mutant allele (A) is very frequently present in gaited breeds and in breeds used for harness racing whereas the breeds of horses typically used for dressage are homozygous for the wild-type allele (C).

Dressage is an Olympic equestrian sport in which horses perform a predetermined test consisting of different gaits and patterns performed in a 20 × 60 m arena. Dressage horses are taught to perform their natural gaits through a wide speed range, thus over-riding the natural trigger to make a transition. As training progresses, the dressage horse learns to move in an uphill posture with the forehand raised and the haunchs lowered. Highly trained dressage horses may learn to perform passage and piaffe, which are regarded as artificial gaits. In nature, horses sometimes perform a display gait similar to passage but this can be distinguished from the competition gait by the fact that the horse’s back is hollow and the body is held stiffly without the roundness and relaxation required in dressage. Biomechanical analysis of different aspects of the dressage horse’s performance of enhanced natural gaits and artificial gaits offers an opportunity to study the range of biomechanical abilities of the athletic horse.

Equine gaits are recognized according to stride kinematics (movements). The walk has a four-beat rhythm with each footfall occurring separately in a lateral sequence; the footfalls may be equally spaced in time or they may occur as lateral or diagonal couplets. Dressage horses typically walk at speeds in the range of 1.4–1.8 m/s [5]. In trot and pace, the movements of a fore and hind limb are synchronized by pairing diagonally or laterally, giving a two-beat rhythm [6,7,8,9]. The speed range for trotting dressage horses has been reported to be 3.2–4.9 m/s [6]. The ambling gaits, performed at intermediate locomotor speeds by horses with the *DMRT3* mutation [4], are lateral sequence gaits that may be coordinated with lateral couplets, diagonal couplets or equal intervals between footfalls [10]. The canter and gallop are asymmetrical gaits in which the hindlimb pair and the forelimb pair move as couplets; the first limb of the couplet to contact the ground is the trailing limb, the second is the leading limb. Horses typically canter and gallop with the leading limb on the same side in the fore and hind limb pairs [11,12]. The range of cantering speeds for dressage horses has been reported to be 3.3–6.0 m/s [11].

Regardless of which gait is being performed, locomotion results from the application of forces that are generated when the hooves press against the ground. Legged locomotion is based on protraction and retraction of the limbs relative to the body. During the retraction phase, the hooves press against the ground to generate ground reaction forces (GRF). The magnitude and orientation of the GRF vector, respectively, determine the speed and direction of body movement. Ground reaction forces are measured using a force plate, which is a metallic plate embedded in the ground. The 3-dimensional GRF vector is resolved into three components acting in the vertical, longitudinal and transverse directions. The vertical component acts in opposition to gravity and provides upwards movement. The longitudinal component controls speed and the transverse component produces turning or lateral movements. The total GRF, which is the summation of the individual GRFs of all concurrently loaded limbs, has gait specific patterns.

Movements of the limbs and body are driven by muscular contractions fueled by the conversion of chemical energy into mechanical energy in the muscle fibers. Minimization of energy expenditure is a prime consideration in neuromotor control of locomotion and one of the ways in which this is accomplished is through conversions between potential energy (PE), kinetic energy (KE) and elastic energy (EE), which are different types of mechanical energy. Efficient conversions between these energy types reduces the need for the muscles to generate mechanical energy de novo. 

Kinetic energy is energy due to movement and is calculated in vertical and longitudinal directions as KE = 1/2mv^2^, where m is the horse’s mass and v is velocity. Potential energy is energy due to position (height) and represents the energy input necessary to raise the body. It is calculated as PE = mgh, where m is the horse’s mass, g is the gravitational acceleration (9.81 m/s^2^), and h is the height of the center of mass (COM). At any instant in time, a body’s mechanical energy is the summation of its KE and PE [13].

Two important methods of conserving mechanical energy during locomotion involve inverted pendulum mechanics and spring mass mechanisms. In inverted pendulum mechanics, out-of-phase changes in height and velocity of the COM facilitate exchanges between PE and KE. In spring-mass mechanics, mechanical energy is temporarily stored as EE in musculoskeletal tissues when both PE and KE decrease during limb loading. The stored EE is released as the elastic tissues recoil during unloading [13].

Biomechanists classify gaits into two broad categories of walking gaits or running gaits based on spatiotemporal kinematics [14,15,16], shape of the vertical GRF curve [17,18], and energy-conservation mechanics [14,15]. Based on spatiotemporal variables, gaits that have an aerial (or suspension) phase in which the horse loses contact with the ground are classified as running gaits, those without an aerial phase are walking gaits [14,15,16]. However, running gaits may lose their aerial phases at slow speeds without a fundamental change in the nature of the gait [14]. The shape of the vertical GRF curve is usually biphasic in walking gaits and monophasic in running gaits [15]. Mechanical energy is conserved using inverted pendulum mechanics in walking gaits or by using spring-mass mechanics in running gaits [13].

Some gaits fit within the same category of walk or run according to all three classification criteria whereas others are assigned differently [15]. The walk has no aerial phase, it has a biphasic vertical GRF curve, and it conserves mechanical energy by inverted pendulum mechanics. Therefore, it is classified uniformly as a walking gait. The trot has aerial phases, a monophasic vertical GRF curve, and it conserves energy using spring-mass mechanics. It is classified as a running gait by all three methods. The tölt, which lacks an aerial phase, combines walking kinematics with a monophasic vertical GRF curve and spring-mass mechanics; it is classified as a running gait with walking kinematics [15,19,20].

A fundamental difference between bipedal and quadrupedal locomotion is that in bipeds the body’s COM is positioned above the limbs and its movements are determined by forces generated by a single pair of limbs located beneath the COM. In a quadruped the COM lies between the fore and hind limbs and its movements are influenced by gravitational and inertial forces on the trunk as well as by GRFs transmitted through the two limb girdles, which differ from each other in timing, magnitude and direction. The proximity to the forelimbs is reflected in a relatively greater influence on COM movements [15,21]. 

The fore- and hind limbs differ structurally and functionally; the strut-like forelimbs are better-designed for inverted pendulum mechanics while the more angulated hind limbs are more easily compressed in a spring-like manner. It is advantageous to evaluate the functions of the forelimbs and hind limbs separately as well as in combination in order to understand how the individual limbs influence whole body mechanics.

This article reviews equine gait classification based on spatiotemporal variables, vertical GRFs, and energy conservation mechanisms and describes these variables in the diagonally coordinated gaits of dressage horses: collected trot, passage, and piaffe.

## 2. Diagonally Coordinated Gaits of Dressage Horses

Dressage horses perform a range of natural and artificial gaits without consideration for energetic efficiency; indeed, an enhanced level of energy expenditure produces more extravagant movements that are rewarded by the scoring system. Dressage horses are taught to walk, trot and canter at an expanded speed range while maintaining a posture in which the forehand is elevated relative to the haunches and the neck is raised and rounded with the poll as the highest point. In contrast to unrestrained locomotion, transitions between gaits are performed in response to a cue from the rider. The following section summarizes the biomechanical characteristics of trot, passage and piaffe and provides definitions of each gait according to the Fédération Equestre Internationale (FEI) rules for dressage [22].

Trot: a two (2)-beat pace of alternate diagonal legs (left fore and right hind leg and vice versa) separated by a moment of suspension. The trot should show free, active and regular steps. The quality of the trot is judged by general impression, i.e., the regularity and elasticity of the steps, the cadence and impulsion in both collection and extension. This quality originates from a supple back and well-engaged hindquarters, and by the ability to maintain the same rhythm and natural balance with all variations of the trot. In collected trot, the horse, remaining “on the bit”, moves forward with the neck raised and arched. The hocks, being well-engaged and flexed, must maintain an energetic impulsion, enabling the shoulders to move with greater mobility, thus demonstrating complete self-carriage. Although the horse’s steps are shorter than in the other trots, elasticity and cadence are not lessened. Working trot is a pace between the collected and the medium trot, in which a horse’s training is not yet developed enough and ready for collected movements. The horse shows proper balance and, remaining “on the bit”, goes forward with even, elastic steps and good hock action. The expression “good hock action” underlines the importance of an impulsion originating from the activity of the hindquarters [22].

Passage: a measured, very collected, elevated and cadenced trot. It is characterized by a pronounced engagement of the hindquarters, a more accentuated flexion of the knees and hocks, and the graceful elasticity of the movement. Each diagonal pair of legs is raised and returned to the ground alternately, with cadence and a prolonged suspension. In principle, the height of the toe of the raised forefoot should be level with the middle of the cannon bone of the other supporting foreleg. The toe of the raised hind foot should be slightly above the fetlock joint of the other supporting hind leg [22].

Piaffe: a highly collected, cadenced, elevated diagonal movement giving the impression of remaining in place. The hindquarters are lowered; the haunches with active hocks are well engaged, giving great freedom, lightness and mobility to the shoulders and forehand. Each diagonal pair of legs is raised and returned to the ground alternately, with spring and an even cadence. In principle, the height of the toe of the raised forefoot should be level with the middle of the cannon bone of the other supporting foreleg. The toe of the raised hind foot should reach just above the fetlock joint of the other supporting hind leg [22].

## 3. Spatiotemporal Variables

Spatiotemporal variables provide information describing the effect of the neuromotor control system on the movements and coordination patterns that allow an observer to recognize and distinguish different gaits. Since the equine diagonally coordinated gaits are classified as symmetrical gaits, the spatiotemporal variables show left-right symmetry. Velocity and stride length decrease progressively and significantly from collected trot to passage to piaffe and the fact that the velocities are different affects many of the kinematic variables. Passage and piaffe have longer stride durations than collected trot.

### 3.1. Footfall Sequence and Timing

Each gait has a characteristic footfall sequence. Indeed, the spectrum of symmetric and asymmetric gaits can be classified based on speed and relative footfall timings using linear discriminant analysis [19]. The description of trot as a diagonally-synchronized gait implies coordinated protraction and retraction of the diagonal limb pairs. Protraction begins around the time of lift off and continues until late swing. Retraction occupies terminal swing and the entire stance phase. The short period of swing phase retraction, which begins at maximal protraction and ends at hoof contact with the ground, slows the forward velocity of the hoof relative to the ground at contact. In trot, swing phase retraction is considerably longer in the forelimbs (~22% stride duration) than the hind limbs (~5% stride duration) [23] and, as a result, the forelimbs contact the ground with a slower horizontal velocity than the hind limbs [24].

**Figure 1:**
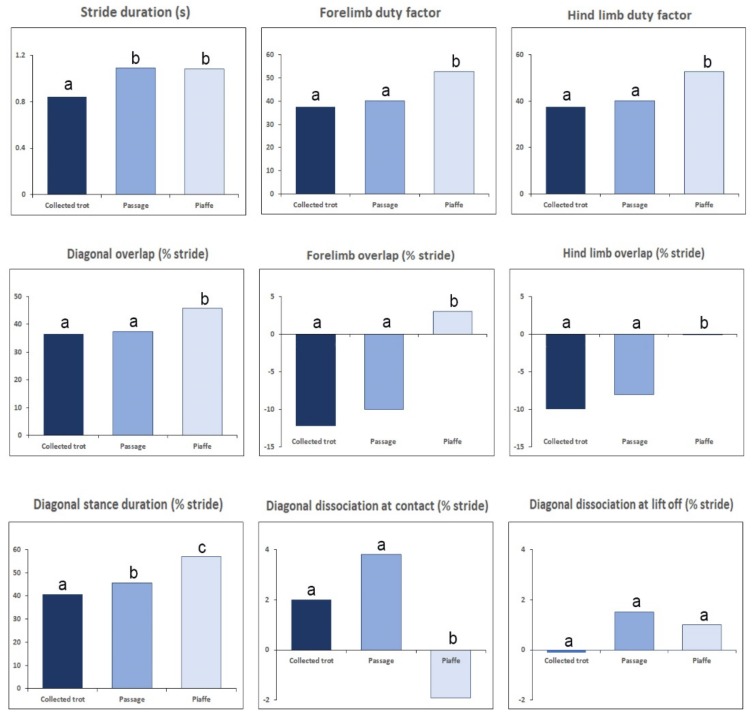
Mean values of temporal variables for collected trot, passage and piaffe [25]. Within each graph, different letters (a,b,c) above the columns indicate values that differ significantly (*p* < 0.05).

Even though the diagonal limb pairs appear to swing synchronously, hoof contacts and lift offs are often slightly dissociated (Figure 1) [7,11,25,26,27,28]. Diagonal dissociation results in short periods of single support at the start and/or end of diagonal stance. Horses trotting in hand at moderate speed (~3.3 m/s) show fore-first, synchronous, and hind-first dissociation patterns that may differ between diagonals [29]. As speed increases the pattern shifts towards a larger number and longer duration of hind-first dissociations and fewer fore-first dissociations [29]. In dressage horses, hind-first contact is associated with higher judged scores for trot quality [27], a more uphill posture [29], a more caudal location of the mean center of pressure (COP) during diagonal stance [29], and lower collisional energy losses [29]. Thus, hind-first contact is regarded favorably. Lift off is almost always hind-first and has not been related to gait quality [29].

Limb contact and lift off times are used to calculate variables, such as duty factor, that affect the possibility of having an aerial phase. In bipeds the equation is simple; if each limb is grounded for less than half of the stride then aerial phases are present. In quadrupeds the situation is complicated by the presence of overlaps between hind and forelimbs [16].

### 3.2. Footfall Sequence and Timing

The duty factor expresses stance duration as a percentage of stride duration (Figure 1). In bipedal locomotion, if duty factor is >0.5 in both legs it implies overlap between contralateral limb stance phases, which precludes the intervention of aerial phases. In quadrupeds, duty factors >0.5 in the fore or hind limb pair preclude having an aerial phase. When duty factor is <0.5, coordination between the hind and forelimbs determines whether an aerial phase is present. Duty factor decreases with speed [30] and is sometimes used as a proxy for speed. It also changes with training [31]; as muscular strength improves, the trot becomes less grounded, and duty factor decreases. In dressage horses, duty factors for medium and extended trot are significantly smaller than for collected and working trot [6].

### 3.3. Limb Phase

Limb phase is a dimensionless metric describing inter-limb coordination patterns in terms of the fraction of the stride that elapses between footfalls of a hind limb and the ipsilateral forelimb [32]. It can be expressed as a value from 0 to 1 where 1 is stride duration or as a percentage of stride duration. In laterally-synchronized gaits, such as the pace, lateral limb phase is close to zero (range: −0.06 to 0.06). In diagonally-synchronized gaits, such as trot, limb phase is close to 0.50 (range 0.44 to 0.56) [16]. Hind-first diagonal dissociation increases the value of limb phase, fore-first dissociation decreases it.

### 3.4. Limb Support Sequence 

The limb support sequence describes the successive limb combinations that support the body during a stride. In diagonally-coordinated gaits the majority of the stride is spent in diagonal support (Figure 1). Since the supporting limbs are positioned cranial and caudal to the transverse body axis and left and right of the longitudinal body axis, diagonal support stabilizes the body against pitch and roll rotations [33].

### 3.5. Aerial Phases

An aerial phase occurs when all limbs are in the swing phase simultaneously, so the horse is airborne and the gait has a bouncing quality. In a trotting horse, if duty factor is <0.5 in both the fore and hind limbs and movements of the diagonal limbs are tightly synchronized with only a short period of diagonal dissociation, there is a possibility of having aerial phases. In trot, both the presence and duration of aerial phases increases with speed [29]. At very slow speeds, however, as in the western pleasure jog (speed ~1.13 m/s), duty factors are high (forelimbs: 0.68; hind limbs: 0.61) and there are no aerial phases [34].

### 3.6. Kinematics of Collected Trot, Passage and Piaffe

**Figure 2:**
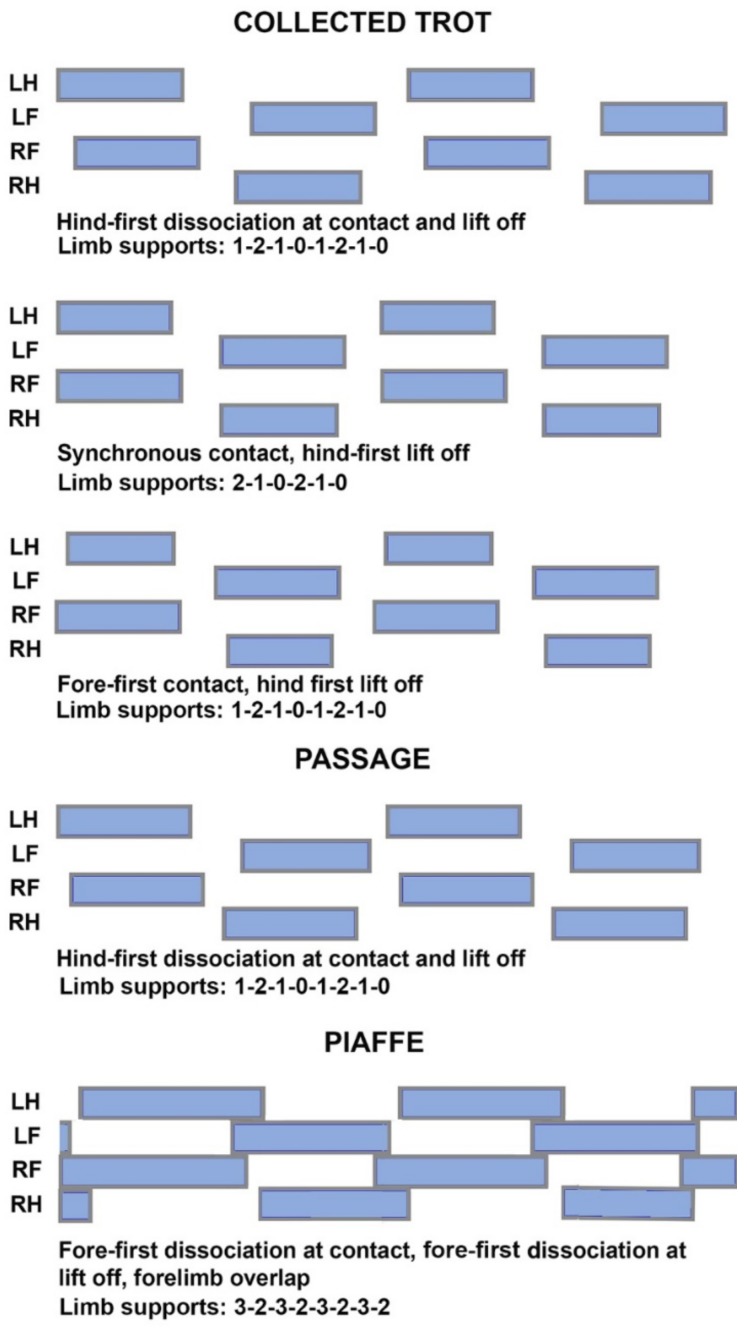
Common limb support sequences for collected trot (**top 3 diagrams**), passage (**middle diagram**) and piaffe (**bottom diagram**). Limb support sequences other than those shown do occur, especially in piaffe. LH: left hind limb; RH: right hind limb; LF: left forelimb; RF: right forelimb.

In dressage horses, the ability to bounce off the ground into a well-defined suspension phase in trot is favorably regarded. In collected trot performed at an average speed of 2.3 m/s [6], diagonal support phases typically alternate with short aerial phases. Hind-first dissociation with limb phase >0.50 is favored since this tends to be associated with nose-up trunk orientation, which is rewarded by the judges. Conversely, fore-first dissociation with limb phase <0.50 and nose-down trunk posture, also described as being on the forehand, receives lower scores. Limb phase has been reported to be 0.50 in dressage horses ridden at collected trot on a treadmill [28], compared with 0.52 overground [25,27]. Duty factors in the range of 35–42% have been reported for collected trot with higher values on a sand surface [25] than on hard-packed gravel [27]. Support sequences for collected trot vary according to the type of diagonal dissociation at contact and lift off (Figure 2).

**Figure 3:**
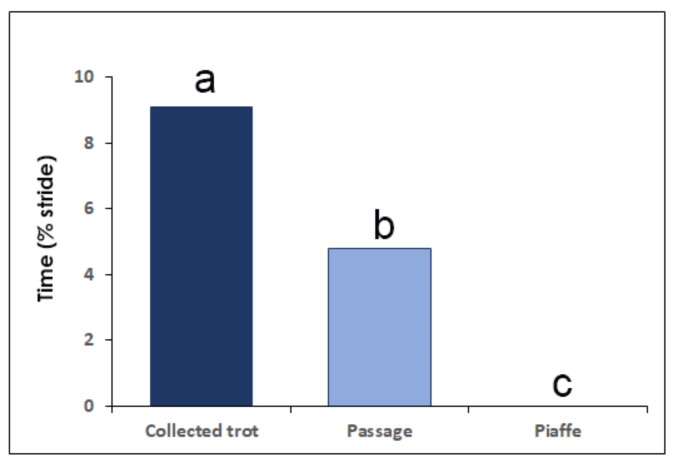
Mean values of aerial phase duration expressed as percentage of stride duration in collected trot, passage and piaffe [25]. Different letters (a,b,c) above the columns indicate values that differ significantly (*p* < 0.05).

Aerial phases are typically present in collected trot with mean duration of 76 ms, equivalent to 9.1% stride duration (Figure 3) [25]. The most common formula for the number of supporting limbs in collected trot is 1-2-1-0-1-2-1-0 when there is diagonal dissociation at contact or 2-1-0-2-1-0 when the fore and hind limbs make synchronous contact.

Passage is a slow, diagonally synchronized artificial movement in which stance phases of the diagonal limb pairs: (left fore-right hind (LF-RH) and right fore-left hind (RF-LH)) alternate with aerial phases. It is distinguished from collected trot by being performed at a slower speed in the range of 1.2–1.9 m/s [35,36,37], with a shorter stride length and a longer stride duration (Figure 1). It also has greater cadence and more pronounced elevation of the limbs which appear to pause at the moment of highest elevation before being lowered. Passage steps show greater kinematic variability than trot steps, which reflects the difficulty of maintaining balance on a diagonal pair of limbs while moving slowly.

Passage usually has hind-first dissociation at contact and lift off [35,37] with dissociation time at contact averaging 30 ms (3% stride duration) when moving overground [27] or on a treadmill [28]. The dissociation times at lift off have large coefficients of variation (around 100%), which reflect step-to-step variability associated with the challenges of maintaining balance at slow speed [37]. Limb phase is in the range of 0.52–0.53 [25,27,28]. The most common limb support sequence has a hind limb in single support, followed by diagonal overlap and a short period of forelimb single support prior to the aerial phase (Figure 2) [28,35]. The aerial phases have shorter absolute (52 ms) and relative (4.8%) durations than collected trot [25]. The sequence of supporting limbs is the same as in collected trot: 1-2-1-0-1-2-1-0.

Piaffe is a diagonally synchronized movement showing the highest degree of collection and self-carriage. At the Grand Prix competition level, piaffe is performed in place. However, a little forward progression, maximally 10 cm per step, is allowed at the Intermediate competition levels. If the horse moves forwards too much or even slightly backwards, it is regarded as a serious fault [22]. Balance is heavily dependent on static equilibrium in piaffe; step-to-step adjustments in the timing and position of the footfalls are made based on feedback information. This results in high variability of the stride variables. 

The majority of piaffe steps show fore-first dissociation [25,35] and, in some horses, dissociation is so long that they perform piaffe as a four-beat gait with a lateral (walking) footfall sequence. Hind-first and synchronous contacts are recorded less frequently. In Olympic competitors, higher-ranked horses were more likely to have synchronous or hind-first dissociation compared with lower-ranked horses [25]. The propensity for fore-first dissociation is reflected in an average limb phase of 0.46 in national level competitors [27] whereas Olympic competitors had an average limb phase of 0.5 [25]. The lift off sequence also varies between and within horses.

Piaffe has significantly higher duty factors than passage or collected trot, resulting in longer limb overlaps that provide a larger base of support for greater stability (Figure 1). Aerial phases are not a feature of piaffe; instead, weight is transferred from one diagonal pair to the other through periods of overlap between the fore and/or hind limbs [25,35]. Some horses leap from one forelimb to the other or bounce from one hind limb to the other, which can fool the eye into thinking there is an aerial phase when, in fact, there is always at least one hoof on the ground. Limb support sequences include periods of tripedal and even quadrupedal support (Figure 2). 

## 4. Ground Reaction Forces

Ground reaction forces, generated in response to the hoof pressing against the ground during the stance phase, determine the acceleration imparted to the body. The 3-dimensional GRF vector is resolved into three mutually perpendicular components acting vertically, longitudinally and transversely. The vertical and longitudinal GRFs are described below with reference to the trotting gaits.

### 4.1. Vertical Ground Reaction Force

**Figure 4:**
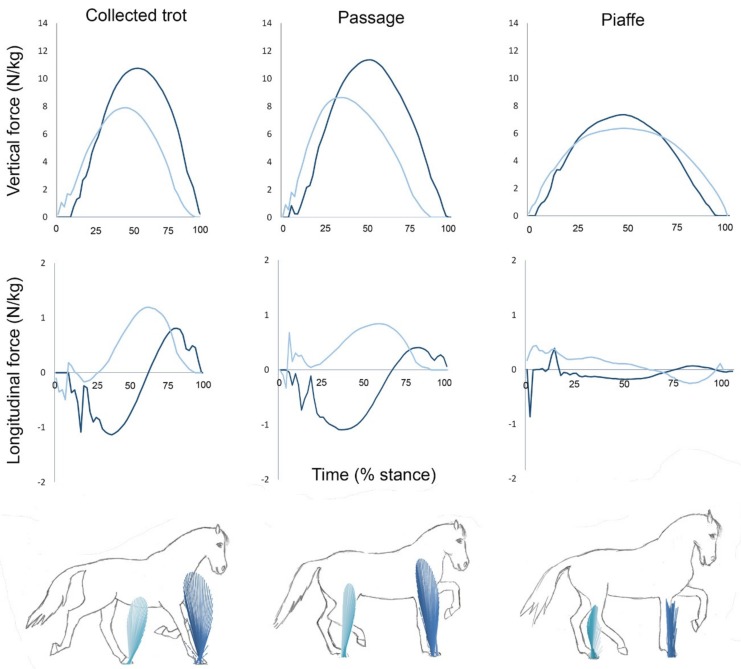
Vertical (**top row**) and longitudinal (**middle row**) ground reaction forces for one horse performing collected trot (**left**), passage (**middle**) and piaffe (**right**). The scale on the y axis is the same for graphs depicting the same force component. The x axis is scaled to the percentage of stance phase but the absolute length is proportional to the absolute stance duration for each gait. Dark lines: forelimb forces; light lines: hind limb forces. The force vector diagrams (**bottom row**) illustrate successive sagittal plane ground reaction force vectors for the fore and hind limbs in each gait.

The vertical GRF component represents gravitational and inertial forces transmitted through the horse’s limbs that are responsible for accelerating the horse vertically, for example during jumping or into a lofty aerial phase. In each gait the vertical GRF curve in the fore and hind limbs has a typical shape though the magnitude may change with speed [38]. For example, at slow walk the vertical GRF curve is broad and flat, at moderate walking speed it is distinctly biphasic, at fast walk the dip between the two peaks is exaggerated, and in trot the two peaks merge into a single peak [18,39,40]. Typically, the vertical GRF curve is biphasic in walking gaits and monophasic in running gaits. For horses trotting in hand the vertical force-time graph rises smoothly, peaks in midstance (Figure 4), then decreases with a small change in slope around 85% stance that represents heel lift. Peak vertical GRF occurs a little earlier in the hind limbs (43% stance) than the forelimbs (45% stance). Peak values do not differ between contralateral fore or hind limbs but are higher in the forelimbs than the hind limbs. Peak total vertical GRF coincides with the forelimb peak, at which time the forelimbs contribute 54% of the total vertical GRF [41].

Vertical impulse is the summation of the vertical GRF throughout the stance phase and is calculated as the area under the vertical GRF curve. Total vertical impulse carried by the forelimbs is 56% for Dutch Warmbloods trotting in hand [40], 57% for ponies trotting in hand [42], and 57% for dressage horses ridden in collected trot [43]. As trotting speed increases from 2 m/s to 5 m/s, peak vertical GRF increases in the forelimbs but not in the hind limbs. Since the forelimbs have a relatively larger reduction in stance duration than the hind limbs, the distribution of the vertical impulse does not change with speed [42,43].

The body center of pressure (COP) is the point of application of the resultant ground reaction force vector. It is calculated by summation of the vertical GRFs of all concurrently loaded limbs. It is important to distinguish between the trajectory of the COM, which represents the movements of the body versus the COP, which represents the point of application of the GRF. The COM and COP follow different paths during locomotion; the COM moves forward constantly, the base of support changes periodically, and the COP moves within the base of support in accordance with the vertical GRF distribution between the grounded limbs. At trot, the COM path is sinusoidal with its height and forward velocity changing in response to the vertical and longitudinal GRFs, respectively. 

The position of the COP reflects the relative contributions of the forelimbs and hind limbs to the total vertical GRF; if the forelimb contribution increases relative to that of the hind limb, the COP moves closer to the forelimbs. In the standing horse, 57.5% of the vertical GRF is borne by the forelimbs and 42.5% by the hind limbs [43] so the COP is located at 42.5% of the distance from the forelimbs toward the hind limbs. During locomotion the force ratio between the grounded fore and hind limbs changes over the stride. In the trotting horse, the COP is initially located beneath the first hoof of the diagonal pair to contact the ground or between the diagonal limbs if contact is synchronous. It moves to a position closer to the forelimbs where it remains in an almost stationary position through mid-stance before moving cranially as the forelimb’s contribution to the vertical GRF increases during the final one third of stance [41].

The position of the COP relative to the COM is important in maintaining balance because it determines the length of the moment arm of the resultant vertical GRF. When the COP is ahead of the COM, the resultant vertical GRF tends to rotate the trunk in a nose-up direction and vice versa if the COP is caudal to the COM [29,37]. Adjustments in the magnitude of the resultant GRF and the length of its moment arm are used to control pitch (nose-up/nose-down) stability. During the middle part of stance, the moment arm of the resultant GRF vector is increasing and this coincides with the time when the hind limb makes its largest contribution to the moment around the COM.

### 4.2. Longitudinal Ground Reaction Force

The longitudinal GRF curve for trot shows an initial braking (negative) phase characterized by large impact spikes in the first 20–50 ms followed by a propulsive (positive) phase in all limbs. When diagonal dissociation is present, the longitudinal forces of the diagonal limb pair are somewhat out-of-phase, which has the effect of reducing the total (summed over fore and hind limbs) magnitudes of the impact spikes, braking GRF peak and propulsive GRF peak as well as decreasing the longitudinal accelerations of the body [41]. When moving at constant speed within a range of moderate trotting speeds, peak propulsive GRF increases with speed in both the fore and hind limbs but the longitudinal impulses are unchanged [42]. The braking impulse is larger in the forelimbs whereas the propulsive impulse is larger in the hind limbs [41].

For horses trotting in hand the transition from braking to propulsion occurs earlier in the hind limbs (around 38% stance) than in the forelimbs (around 53% stance), so there is a period in mid-stance when the forelimbs are braking while the hind limbs are propelling. During this time, the sagittal plane GRF vectors converge [37]. This has been suggested to be a mechanism to enhance pitch stability [44].

### 4.3. Ground Reaction Forces of Collected Trot, Passage and Piaffe

In collected trot the GRFs conform, in most respects to the general description for the trot. At the start of diagonal stance the COM is located at 78% of the diagonal length behind the forelimb. It moves forward at fairly constant velocity to a position just ahead of the fore hoof when it lifts off. 

The COP is initially located beneath the first hoof to make contact. After some initial oscillations it becomes almost stationary at about 45% of the diagonal distance behind the forelimb and remains in this position through the middle part of stance. In the second half of stance, as the forelimb contribution to total vertical GRF increases, the COP moves cranially until it is located at the level of the forelimb during its period of single support in terminal stance. 

The COM is initially caudal to the COP, becoming coincident with it at around one third of diagonal stance duration, then moving further ahead of the COP until around two thirds of diagonal stance duration. At this time the increasing forelimb vertical GRF causes the COP to move forward and become closer to the COM in late stance [41]. 

Passage has monophasic vertical GRF curves in the fore and hind limbs. Peak vertical forces are not different from those of collected trot but passage has significantly higher vertical impulses in both fore and hind limbs as a result of having longer stance durations. All four limbs contribute to the increased vertical impulse that is responsible for the larger vertical oscillations of the COM in passage, however the hind limbs make a relatively greater contribution (+34%) than the forelimbs (+17%) [45]. Compared with collected trot, passage has a significantly earlier transition from braking to propulsion and a higher propulsive impulse, which is partly a result of the prolongation in hind limb stance duration [45]. The time during which the fore and hind limb sagittal plane GRF vectors converge is longer in passage than collected trot.

In passage, the COM is initially located at approximately 70% of the diagonal distance from the fore hoof at the start of stance then progresses forward to a position 20–40% of the diagonal distance behind the fore hoof at lift off. Thus, the COM does not move as far forward relative to the base of support in passage compared with collected trot. The COP is initially located beneath the hind hoof, which is the first of the diagonal pair to contact the ground. It moves forward gradually and, in the middle part of stance, it tracks the COM quite closely. The proximity of the COP to the COM is achieved by adjusting the relative contribution of the vertical GRFs between fore and hind limbs. The benefit to having the COP almost coincident with the COM is that it minimizes the moment arm of the resultant vertical GRF [37].

Piaffe has considerably longer stance durations in both fore and hind limbs than collected trot or passage [25,35] which allows more time to generate the necessary impulses to perform the movement, so peak vertical forces are low. The shape of the curve is monophasic. Unlike the trot and passage, in which the longitudinal GRFs control the forward movement of the COM relative to the diagonal base of support [37,41], in piaffe the COM should either be stationary or progress only slightly forward. This implies that either the GRF vectors are vertical throughout stance or the longitudinal forces in the fore and hind limbs are balanced to minimize accelerations of the COM. The GRF curves for piaffe in Figure 4 show the hind limbs exerting a small propulsive force that is balanced by a small braking force in the forelimbs through the first 70% of stance duration after which the force direction changes in both limbs. The opposing longitudinal forces in the fore and hind limbs allow the horse to piaffe on the spot. The ability to generate an appropriate GRF profile and adjust the fore:hind vertical GRF ratio is essential for controlling moments around the horse’s COM and maintaining balance in piaffe.

## 5. Gait Mechanics

During locomotion, mechanical work is used to move the body segments relative to the body and to move the body relative to the environment. The neuromuscular control system aims to minimize the work done through the use of energy-conservation strategies that involve exchanges between different types of mechanical energy primarily through the use of inverted pendulum mechanics or spring-mass mechanics [13]. The benefit of energy conservation is that it minimizes the amount of muscular work required to drive the movements of the limbs and body and, thus, reduces the metabolic cost of locomotion. 

Evaluation of the COM trajectory and the associated energy fluctuations during the stride is regarded as the gold standard for differentiating walking vs. running gaits. In a walking gait, PE and KE oscillate out-of-phase, whereas in a running gait, PE and KE oscillate in-phase [13]. 

### 5.1. Inverted Pendulum Mechanics

Inverted pendulum mechanics is used in walking gaits. The underlying principle is that out-of-phase changes in COM height and velocity allow exchanges between PE and KE during the stance phase [13,14]. This type of exchange is possible in the walk because the relationship between limb stiffness and vertical GRF is such that the body vaults up and over the foot during the middle part of stance. 

Idealized inverted pendulum mechanics apply quite well in biped species in which both legs are located below the COM. The COM is low at the start of stance, ascends as it vaults upward over a relatively stiff leg during the middle part of stance and is low again at the end of stance. The stance phase begins and ends with a short period of overlap between contralateral legs and it is during the intervening single support phase that the leg is stiffened, allowing it to act as a strut that rotates over the grounded foot. Potential energy increases as the COM rises in the first half of single support. At the same time the forward and upward velocities of the COM are slowing and KE is decreasing in both vertical and longitudinal directions. As the COM accelerates downward and forward in the second half of stance, the changes in PE and KE are reversed. The out-of-phase changes in KE and PE allow energy to be exchanged between them thus conserving the body’s mechanical energy and reducing the need for muscle work [14].

In quadrupeds, the fore and hind limbs operate somewhat independently and behave like two bipeds moving in series. The forelimbs support the withers, the hind limbs support the croup, and the trunk segment containing the COM is suspended between them. Thus, COM oscillations are affected, not only by the movements of the forelimbs and hind limbs but also by the coordination between them, as well as by movements of the thoracolumbar spine and the ribcage. The stride-by-stride range of sagittal plane motion of the equine COM during trotting was shown to be smaller than that of T16 by 27% vertically and 24% craniocaudally [46]. Thus, movements of the COM do not necessarily follow those of markers located on the dorsal midline and there is value in comparing the excursions of the withers, croup, and COM in order to understand the complexities of quadrupedal energetics.

In walking horses, the limbs of the pelvic and thoracic girdles show inverted pendulum mechanics, but as a consequence of the sequential footfall timings in the walk, there is an offset in the exchanges between PE and KE in the hind limbs and forelimbs. In dressage horses, the footfalls should be equally spaced through the stride so limb phase is 0.25, though many horses deviate from this rhythm [5]. If limb phase is 0.25, fluctuations between PE and KE are out-of-phase in the hind and forelimbs. As PE increases and KE decreases in early forelimb stance, the reverse changes are occurring in the ipsilateral hind limb, then as PE decreases and KE increases in late forelimb stance, the reverse changes occur in the diagonal hind limb. Changes in longitudinal KE coincide with changes in longitudinal GRFs; Merkens and Schamhardt [47] observed that during walking, no more than one limb provided either a braking or a propulsive longitudinal force at any given time. 

The relative timing of the fore and hind limb movements has a large influence on COM motion and the consequent energy exchanges [15]. In a dog model with ipsilateral limb phase 0.25 and equal weight distribution between the fore and hind limbs, the COM had a flat trajectory with no pendular energy exchanges [48]. The fact that dogs walk naturally with lateral couplets using limb phase around 0.15 and carry more than half their weight on the forelimbs, allows some pendular energy exchange. In collected, medium and extended walks, 67% of dressage horses walked with limb phase <0.25 [5], which may facilitate pendular energy exchanges. 

The downward motion of the COM is reversed during the periods of overlap between contralateral limbs at the start and end of their stance phases. Potential energy decreases gradually as the COM approaches its lowest point. The contralateral limbs have opposing longitudinal GRFs; the protracted limb, which is at the start of its loading phase, exerts a braking GRF while the retracted limb exerts a propulsive GRF as it pushes off into its swing phase. Energy dissipation is reduced when the push off forces in the retracted limb precede the braking forces in the protracted limb [49].

### 5.2. Spring-Mass Mechanics

Spring-mass mechanics are used extensively in running gaits, in which the joints of the limbs flex and limb length shortens during the middle part of stance. Both PE and KE are high at the start and end of stance and decrease to a minimum in mid-stance. The fact that PE and KE oscillate in-phase precludes energy exchanges between them [13]. Instead, mechanical energy is stored as EE in tendinous tissues that undergo a stretch-recoil cycle as the joints flex and extend. Control of joint flexions and EE storage may be by eccentric-concentric muscular contractions, isometric muscular contraction combined with stretching and recoil of an elastic tendon, or a stretch-shorten cycle in an elastic ligament. Thus, mechanical energy is exchanged between KE, PE and EE as the limbs compress and lengthen during stance. Measuring PE and KE is relatively easy but calculating EE exchanges is more difficult [13].

When using spring-mass mechanics, the action of the muscles is initially to slow the downward and forward motion of the body then to push the body up and forward often, though not always, into an aerial phase. In general, the proportion of step duration spent in the aerial phase is 0–15% at trot, 0–30% at gallop and 0–75% during hopping [13].

### 5.3. Center of Mass Mechanics in Collected Trot, Passage and Piaffe

**Figure 5:**
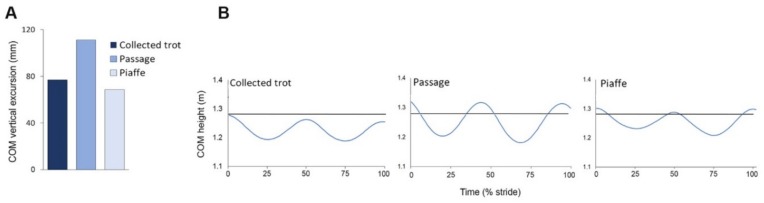
(**A**) Vertical excursion of the center of mass per stride during collected trot, passage, and piaffe. (**B**) Height of the center of mass during a complete stride starting and ending at hind contact for collected trot (**left**), passage (**middle**) and piaffe (**right**). The horizontal line represents standing height of the center of mass (COM).

In all three gaits the COM follows a sinusoidal pattern in which the maxima occur around the time of weight transfer between diagonals and the minima occur in midstance (Figure 5). Oscillations of the COM are largest in passage and are of similar, but smaller, magnitude in collected trot and piaffe even though piaffe has no aerial phases (Figure 5).

**Figure 6:**
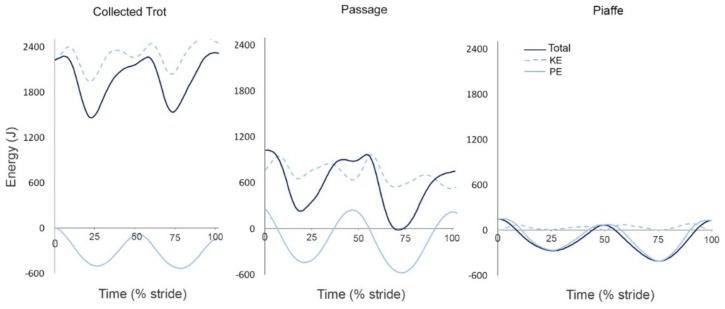
Changes in total mechanical energy (solid dark line), total kinetic energy (KE, dashed light line) and potential energy (PE, solid light line) during one stride of collected trot (**left**), passage (**center**) and piaffe (**right**). The data are for one stride, starting and ending at hind contact. The scale on the y axis is the same for all graphs. The x axis is scaled to percentage of stride but its length is proportional to the absolute stance duration for each gait. Values for KE are absolute. Values for PE are reported as changes compared to standing.

Changes in PE follow the same pattern and have similar timing to the vertical oscillations of the COM with two peaks per stride in all three diagonally synchronized gaits (Figure 6). Both vertical and longitudinal KE oscillate with a frequency double that of the PE fluctuations because they are based on changes in velocity rather than displacement of the COM in vertical and longitudinal directions, respectively. At diagonal contact the COM is accelerating downward. The downward velocity slows under the influence of the vertical GRF and is zero at the time of maximal vertical GRF. The COM is then accelerated upward. The upward vertical velocity of the COM increases then decreases to zero around the time of lift off [41]. Longitudinal KE also has four cycles per stride; there is a large decrease in longitudinal KE due to the effects of the braking longitudinal GRF in the first half of diagonal stance followed by a smaller decrease coinciding with the aerial phase when GRFs are absent. Total KE (Figure 6) reflects the changes in both vertical and longitudinal KE.

In passage, PE and vertical KE follow the same pattern as in collected trot but with larger excursions corresponding with the larger COM vertical range of motion while longitudinal KE has considerably lower values than in collected trot due to the slow forward speed of passage. Total energy is also correspondingly smaller in passage (Figure 6).

In piaffe, PE follows the same biphasic pattern but with smaller oscillations than in the other gaits and KE is very small due to the lack of forward motion combined with the small vertical oscillations. Consequently, total energy is similar to PE throughout the stride (Figure 6).

### 5.4. Equine Limb Mechanics

In running bipeds, the COM is high at the start of stance, descends to its lowest point in midstance, then rises again in late stance [13]. In quadrupeds, compression of the hind limbs and forelimbs occur somewhat independently with the possibility of differences in magnitude and in time of occurrence. Limb compressions are assessed from changes in height of the croup and the withers, respectively. Oscillations of the COM follow the same general pattern as the withers and croup but are likely to differ in magnitude and may show a temporal offset. 

**Figure 7:**
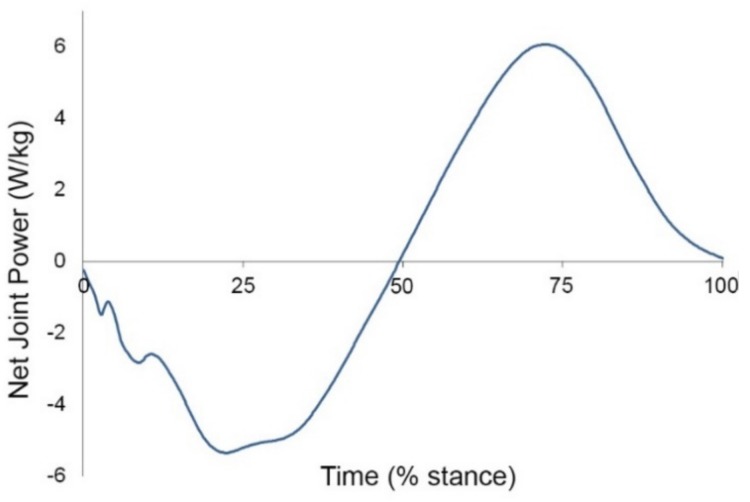
Net joint power curve for the fore fetlock joint at trot. Negative values indicate energy absorption, positive values indicate energy generation. The areas under the negative and positive parts of the curve indicate similar amounts of energy absorption in early stance and energy generation in late stance which is typical of elastic energy storage and release in the soft tissues [54].

Horses are well-endowed with spring-like anatomical structures capable of EE storage and release. These include, but are not limited to, tendons and ligaments in the limbs, such as the biceps brachii tendon, peroneus tertius, superficial digital flexor tendon, suspensory ligament, and fascial tissues in the limbs and trunk [50,51,52,53]. Inverse dynamics analysis can be used to calculate net joint moments and net joint powers across the joints. When a joint is supported by elastic tissues the net joint power curve shows a typical pattern of energy absorption in early stance followed by a similar amount of energy generation in late stance. This pattern is seen across several of the horse’s joints during trotting, notably the fetlock which hyperextends as it is loaded in early stance under control of the digital flexor tendons and the interosseous ligament on the palmar side of the joint. Subsequently, the supporting elastic tissues recoil as the fetlock flexes in late stance. These movements are associated with a net joint power curve showing almost equal amounts of elastic energy storage and release (Figure 7) [54].

Conformationally, the hind limb is particularly well-suited to compress and store elastic energy during stance [42]. Indeed, the hind limbs have been shown to provide about two-thirds of the total elastic energy storage both in running [55] and ambling [15] gaits. The forelimbs are better-suited to use inverse pendular dynamics than the hind limbs because when the carpal joints lock into the close-packed position at the start of stance [56] the entire forelimb acts as a strut over which the withers are vaulted. Even though inverse pendulum mechanics is based on having a stiff limb during stance, this does not preclude some elastic limb compression in mid-stance that reduces the oscillations in KE and PE in walking gaits [57]. In other words, even when the carpal joint is locked in extension, the shoulder, elbow, fetlock and coffin joints can still contribute to forelimb shortening [56]. 

The presence of an aerial phase is not essential for the use of spring-mass mechanics as shown by the ambling gaits [15,20]. For example, tölt has duty factors and dimensionless speed typical of a running gait but the lack of an aerial phase and percent energy recovery are more akin to walking [20]. Furthermore, during tölting, Icelandic horses have vertical ranges of motion of 70 mm and 66 mm, respectively, in the shoulder and hip, with in phase fluctuations of KE and PE that are compatible with spring-mass mechanics. The COM undergoes relatively small vertical excursions of only 12 mm due to the out-of-phase coordination between fore and hind limbs [15]. 

**Figure 8:**
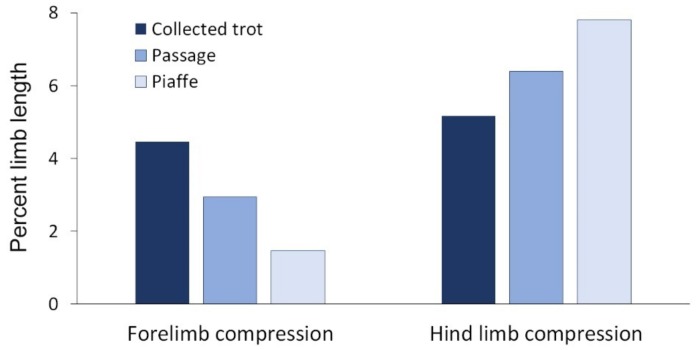
Forelimb and hind limb maximum vertical compression relative to standing height during the stance phase in collected trot, passage and piaffe.

When the limb acts as a spring, overall limb compression can be measured as the difference between the maximal height at the start/end of stance and the minimal height in midstance of the withers or croup. [21] As locomotor speed increases, the limbs sweep through a larger angle during stance, which implies a lower position of the withers and croup at contact and lift off while the stiffness of the limb spring and minimal limb length do not change with speed [58]. The combination of a lower maximal limb height with no change in minimal limb height results in less limb compression at faster speeds and, conversely, greater compression at slower speeds when the limb is more vertical (higher) at contact and lift off. As shown in Figure 8 the amount of hind limb compression relative to standing height increases progressively from collected trot to passage to piaffe [21] and this is consistent with the progressively shorter stride lengths, smaller sweep angles, and higher positions of the withers and croup at the start and end of stance in passage and piaffe [35]. The forelimb, however, shows the opposite changes with progressively less limb compression relative to standing height from collected trot to passage to piaffe.

In trot and passage, both the forelimbs and the hind limbs act as springs, but compared with trot, the hind limbs compress relatively more and the forelimbs relatively less in passage (Figure 8) [21]. Vertical motion of the COM is predicted by length changes of both the forelimb and hind limb during stance [21] with the forelimbs having a relatively greater effect within each gait due to their closer proximity to the COM.

In piaffe, the limbs are raised and lowered without forward progression so there is virtually no sweep of the limbs. When people hop in place at different frequencies, the stiffness of the limb springs changes more than two-fold as a result of changes in knee flexion [58]. Dressage horses are trained to allow the hip, stifle and tarsal joints to undergo greater flexion in the stance phase of piaffe than in the forward-moving diagonal gaits. Collectively, these joint compressions shorten the length of the hind limb and lower the croup. The crouched hind limb posture resembles Groucho running in people [59] in which running with bent knees increases the compliance of the leg spring leading to an increased stance duration and loss of aerial phases while maintaining bouncing mechanics of the COM. At the same time, smaller peak forces allow the forelimbs to act as struts to support the forehand in an elevated position and with less compression than in passage or collected trot (Figure 8). Both hind limb compression and forelimb lengthening contribute to the uphill posture of the horse [21].

## 6. Conclusions

The absence or presence of aerial phases was formerly regarded as distinguishing between walking and running gaits [16,33] but it is now acknowledged that aerial phases alone do not represent functional mechanics [60]. Management of the movements of the COM and the associated exchanges between different types of mechanical energy are recognized as the most important criteria for distinguishing between walking and running gaits. The shape of the vertical GRF curve reflects how forces are used to control COM movements.

In this paper, we have summarized current knowledge of the kinematics, GRFs and energy management strategies for an enhanced natural gait (collected trot) and two artificial gaits (passage, piaffe) performed by dressage horses in the context of classifying them as walking or running gaits. Collected trot conforms to the general pattern of a running gait in having limb phase close to 0.5, duty factor <0.5, and the presence of well-defined aerial phases. During each diagonal step, the graph of the total vertical GRF is monophasic, peaking in midstance when the COM reverses its direction from forward-downward to forward-upward. Height changes of the COM show a regular pattern with peaks during the aerial phases alternating with dips in midstance. Potential energy shows the same pattern and frequency as the COM motion. Vertical KE and longitudinal KE show regular patterns with a frequency twice as high as for PE because they depend on changes in COM velocity rather than height. Overall, changes in KE are partly in-phase and partly out-of-phase with those of PE (Figure 6). In summary, the kinematic, GRF, and mechanical energy exchange patterns are consistent with the classification of collected trot as a running gait by all criteria.

Passage retains the kinematic, GRF and energy exchange characteristics of collected trot (Figure 1, Figure 4, Figure 5. Figure 6) with some differences in magnitude associated with the slower speed of progression and higher swing phase elevation of the COM. Therefore, passage is also classified as a running gait.

In the absence of forward motion, piaffe has some major differences from passage and trot. Kinematically, piaffe has significantly longer duty factors in both the forelimbs and hind limbs and, as a result, has significantly longer overlaps between diagonal limbs and between the contralateral forelimbs (Figure 1, Figure 2). Consequently, there are no aerial phases (Figure 3). The movements of the diagonal limb pairs are less tightly synchronized than in trot or passage and there tends to be greater variability in piaffe steps due to the difficulty of maintaining balance in the absence of forward motion. The COM vertical oscillations and the vertical GRF curve follow the same pattern but have a smaller range of motion than in collected trot or passage (Figure 4, Figure 5); they are consistent with spring-mass mechanics. Piaffe retains a similar pattern of changes in PE and vertical KE but with smaller magnitude than in passage or collected trot. Changes in longitudinal KE are very small as would be expected in a gait performed without forward motion. Oscillations in total KE are small (Figure 6). The hind limbs show more compression and the forelimbs show less compression than in collected trot or passage. Based on these variables, piaffe has the kinematics of a walking gait combined with GRF and COM energy exchanges consistent with running mechanics. 

It is suggested that future research be directed toward comparing the behavior of the forelimbs and hind limbs in relation to the movements and mechanics of the COM, especially in piaffe. Measurements of mechanical variables describing the relationships between PE and KE, such as relative phase, percent congruity and percent recovery will provide further insight into the underlying mechanisms of the gaits and movements of dressage horses.
